# Human Oxygenase Variants Employing a Single Protein Fe^II^ Ligand Are Catalytically Active

**DOI:** 10.1002/anie.202103711

**Published:** 2021-05-19

**Authors:** Amelia Brasnett, Inga Pfeffer, Lennart Brewitz, Rasheduzzaman Chowdhury, Yu Nakashima, Anthony Tumber, Michael A. McDonough, Christopher J. Schofield

**Affiliations:** ^1^ Chemistry Research Laboratory and the Ineos Oxford Institute for Antimicrobial Research University of Oxford 12 Mansfield Road Oxford OX1 3TA UK; ^2^ Present address: Institute of Natural Medicine University of Toyama 2630-Sugitani 930-0194 Toyama Japan

**Keywords:** aspartate/asparagine-β-hydroxylase, biomimetic catalysis, facial triad, metallo-enzymes, 2-oxoglutarate dependent oxygenase

## Abstract

Aspartate/asparagine‐β‐hydroxylase (AspH) is a human 2‐oxoglutarate (2OG) and Fe^II^ oxygenase that catalyses C3 hydroxylations of aspartate/asparagine residues of epidermal growth factor‐like domains (EGFDs). Unusually, AspH employs two histidine residues to chelate Fe^II^ rather than the typical triad of two histidine and one glutamate/aspartate residue. We report kinetic, inhibition, and crystallographic studies concerning human AspH variants in which either of its Fe^II^ binding histidine residues are substituted for alanine. Both the H725A and, in particular, the H679A AspH variants retain substantial catalytic activity. Crystal structures clearly reveal metal‐ligation by only a single protein histidine ligand. The results have implications for the functional assignment of 2OG oxygenases and for the design of non‐protein biomimetic catalysts.

## Graphical abstract

A human 2‐oxoglutarate and Fe^II^ dependent oxygenase was engineered to possess only one protein Fe^II^ binding ligand. The resultant variants retained significant levels of catalytic activity. Biomimetic catalysts containing only one metal binding site are therefore of interest.

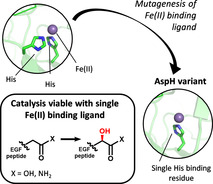

## Introduction

2‐Oxoglutarate (2OG) and Fe^II^ dependent oxygenase superfamily enzymes are widely distributed and catalyse a range of oxidations, typically hydroxylations, though they catalyse other reactions including hydrogenations, desaturations, fragmentations, and ring formations.^[1]^ In humans, they have important physiological roles, for example in the hypoxic response and epigenetics.^[2]^ Most, but not all, of their reactions comprise two electron substrate oxidations coupled to conversion of 2OG to succinate and CO_2_. They have a conserved distorted double‐stranded β‐helix core fold which supports an active site with a single Fe^II^. The consensus mechanism for 2OG oxygenases involves an ordered sequential process in which 2OG, then substrate, then O_2_ bind at the active site.^[3]^ Structural studies have shown many 2OG oxygenases employ a triad of conserved His, Asp or Glu, His residues (the HXD/E…H motif) to ligate the active site Fe^II^, leaving three coordination sites for 2OG, then O_2_ to bind to the Fe^II^, the former via its oxalyl group.[^1a^, ^4^]

**Figure 1:**
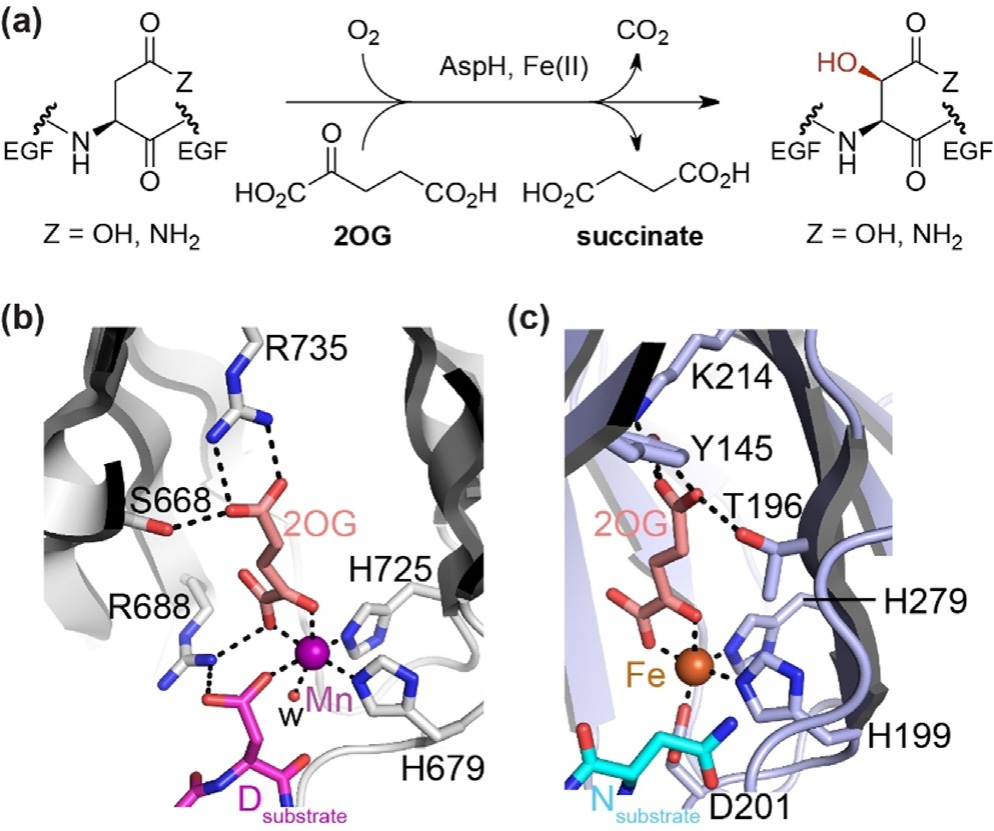
AspH catalyses a typical 2OG oxygenase reaction but has an atypical Fe^II^ binding mode, employing two rather than the typical three protein residues. a) The AspH reaction; b) view of the AspH Fe^II^ binding site (H679 and H725 complex the active site metal ion with Mn substituting for Fe; PDB: 6YYW)^[8]^ compared with that of c) a human 2OG oxygenase with an Fe^II^ binding triad (i.e. H199, H279, and D201; PDB: 1H2L),^[9]^ that is, the asparagine residue hydroxylase, factor inhibiting hypoxia‐inducible transcription factor HIF‐α (FIH).

Aspartate/asparagine‐β‐hydroxylase (AspH) is a human 2OG oxygenase that catalyses C3 hydroxylations of Asp/Asn residues in epidermal growth factor like domains (EGFDs; Figure 1 a)^[5]^—AspH is a proposed target for cancer treatment.^[6]^ AspH is highly unusual amongst structurally characterised 2OG dependent hydroxylases in employing only two residues, H679 and H725, to ligate its Fe^II^ (Figure 1 b).^[7]^ In this regard, it is related to the 2OG dependent halogenases, where the absence of the Asp/Glu residue of the HXD/E…H motif is used to enable Fe^II^ halide binding.[^1b^, ^4^] Here we report the unexpected observation that AspH variants with a single Fe^II^ histidine ligand retain substantial catalytic activity. The results have implications for studying the bioinformatical functional assignment of 2OG oxygenases, which have extensively relied on the HXD/E…H triad, and the design of non‐protein biomimetic catalysts.

## Results and Discussion

Catalytically inactive variants of Fe^II^ ligating residues of the 2OG oxygenases are commonly used in controls during functional assignment work involving cellular studies, including for AspH.^[10]^ As part of our work on AspH, we therefore made the H679A and H725A variants of our previously used human AspH construct (His_6_‐AspH_315‐758_).^[7]^ The requisite mutations were made by standard methods and the variants were purified to near homogeneity by Ni^II^‐affinity and size‐exclusion chromatography (>95 % pure by SDS‐PAGE and MS analysis; Supporting Figure S1). Initially, we conducted turnover assays for the AspH variants alongside wildtype (wt) AspH under reported assay conditions, analysing hydroxylation of a synthetic peptide substrate, hFX‐EGFD1_86‐124_‐4Ser (Supporting Figure S2a),^[7]^ which mimics the requisite disulfide isomer of the EGFD1 of the reported AspH substrate human coagulation factor X (hFX).^[11]^ Unexpectedly, the results revealed that, whilst the variants were less active than wt AspH, they both retained substantial activity: in particular the H679A variant manifested ≈50 % substrate turnover under conditions where wt AspH gave >95 % turnover (30 minutes incubation); the H725A variant was less active (<5 %), but clear evidence for substrate hydroxylation was observed by mass spectrometry (MS) (Supporting Figure S2b). We then carried out more detailed kinetic analyses using our previously reported solid phase extraction coupled to MS (SPE‐MS) assay, monitoring substrate depletion/product formation (+16 Da),^[12]^ under optimized buffer conditions (Supporting Figure S3).

Apparent Michaelis constants ($K_{m}^{\mathrm{app}}$ ) and turnover numbers ($k_{\mathrm{cat}}^{\mathrm{app}}$ ) of wt AspH for Fe^II^ and 2OG are in good agreement with reported data, which were determined using a different synthetic cyclic peptide substrate.^[12]^ The $k_{\mathrm{cat}}^{\mathrm{app}}$ ‐values for the H679A AspH variant are marginally lower than those of wt AspH (Table 1), assuming approximately full enzyme activity as suggested by active site titration of wt AspH.^[12]^ By contrast, the $k_{\mathrm{cat}}^{\mathrm{app}}$ ‐values for the H725A AspH variant are approximately half those for H679A AspH (Table 1), in agreement with the observed lower substrate hydroxylation in our initial turnover assays (Supporting Figure S2b).

**Table 1:** Steady‐state kinetic parameters for the H679A and H725A AspH variants. Apparent turnover numbers ($k_{\mathrm{cat}}^{\mathrm{app}}$ ), apparent Michaelis constants ($K_{m}^{\mathrm{app}}$ ), and specificity constants ($k_{\mathrm{cat}}$ /$K_{m}$ ) of wt, H679A, and H725A His_6_‐AspH_315‐758_ determined for Fe^II^, 2OG, and the hFX‐EGFD1_86‐124_‐4Ser substrate peptide.^[a,b]^

Entry		AspH variant	$k_{\mathrm{cat}}^{\mathrm{app}}$ [s^−1^]	$K_{m}^{\mathrm{app}}$ [μM]	$k_{\mathrm{cat}}$ /$K_{m}$ [mM^−1^ s^−1^]
1	Fe^II[c]^	wt	0.24±0.04	7.2±2.6	33.3±13.2
		H679A	0.18±0.01	4.4±0.8	40.9±7.8
		H725A	0.09±0.01	12.3±3.0	7.3±2.0
2	Fe^II[d]^	wt	0.27±0.04	4.3±1.6	62.8±25.2
		H679A	0.19±0.01	2.7±0.5	70.4±13.6
		H725A	0.10±0.01	8.1±1.8	12.3±3.0
3	2OG	wt	0.31±0.03	1.1±0.4	282±106
		H679A	0.18±0.01	109±20	1.7±0.3
		H725A	0.08±0.01	211±65	0.38±0.13
4	hFX‐EGFD1_86‐124_‐4Ser^[e]^	wt^[f]^	n.d.	n.d.	n.d.
		H679A	0.18±0.01	1.5±0.3	120±24.9
		H725A	0.10±0.02	1.7±1.1	58.8±39.8

[a] Determined using 0.2 μM AspH variant or 0.1 μM wt AspH by SPE‐MS. Detailed analyses are given in Supporting Figures S4–S6. [b] Mean of three independent runs (*n*=3; mean ± standard deviation, SD). [c] Without L‐ascorbate (LAA). [d] With LAA. [e] $K_{m}$ and $k_{\mathrm{cat}}$ values were determined monitoring the hydroxylation of the hFX‐EGFD1_86‐124_‐4Ser substrate peptide (Supporting Figure S2a). [f] Apparent substrate inhibition combined with low detectability of the peptide at low concentrations prevented determination.

Interestingly, the H679A AspH $K_{m}^{\mathrm{app}}$ ‐values for Fe^II^, either in the presence or absence of L‐ascorbate (LAA), and the hFX‐EGFD1_86‐124_‐4Ser substrate peptide are, within experimental error, in the range of those of wt AspH (Table 1) and those reported for other 2OG oxygenases.^[13]^ As for wt AspH,^[12]^ the presence of LAA reduces the $K_{m}^{\mathrm{app}}$ ‐value for Fe^II^, but LAA is not a requirement for productive catalysis by the AspH variants (Supporting Figure S7). Notably, the H679A and wt AspH $K_{m}^{\mathrm{app}}$ ‐values for 2OG differ substantially, the former is about two orders of magnitude higher than the latter (Table 1, entry 3). The slightly different assay conditions (0.2 μM H679A AspH in the absence of NaCl vs. 0.1 μM wt AspH and 50 mM NaCl in the reaction buffer; Supporting Figures S4–S6) likely do not account for this substantial discrepancy. Though higher than the 2OG $K_{m}^{\mathrm{app}}$ ‐values for wt AspH and other human 2OG oxygenases,^[14]^ the 2OG $K_{m}^{\mathrm{app}}$ concentration for H679A is in the range of 2OG concentrations reported in cells (up to >1 mM^[15]^) and lower than the reported 2OG $K_{m}^{\mathrm{app}}$ ‐value for γ‐butyrobetaine hydroxylase (≈150–470 μM).^[16]^

Despite the difference in the $K_{m}^{\mathrm{app}}$ ‐values for 2OG, the results with H679A AspH imply that the presence of only a single polar Fe^II^‐coordinating residue does not preclude efficient hydroxylation—at least in the case of this variant under conditions when 2OG is not limiting. They also reveal the importance of the Fe^II^ chelating histidines in productive 2OG binding.

The H725A AspH substitution appears to have a more pronounced effect than the H679A substitution on the $K_{m}^{\mathrm{app}}$ ‐values for Fe^II^, which are approximately twofold and threefold higher than those of wt and H679A AspH, respectively (Table 1, entries 1 and 2). Strikingly, for H725A AspH, the $K_{m}^{\mathrm{app}}$ ‐value for 2OG is ≈200 times greater than that of wt AspH and about double that of H679A AspH (Table 1, entry 3). By contrast, the H725A AspH $K_{m}$ ‐value for the hFX‐EGFD1_86‐124_‐4Ser substrate peptide is in the range of that of H679A AspH (Table 1, entry 4).

The $k_{\mathrm{cat}}$ /$K_{m}$ ‐values (specificity constants) highlight the large discrepancies between wt AspH and the two variants with respect to 2OG (Table 1). The H679A and H725A AspH $k_{\mathrm{cat}}$ /$K_{m}$ ‐values for 2OG are substantially smaller than that of wt AspH, whereas the $k_{\mathrm{cat}}$ /$K_{m}$ ‐values for Fe^II^ are, within error, similar. NMR turnover assays in the absence of substrate show that H679A and H725A AspH‐catalysed oxidative decarboxylation of 2OG is highly coupled to substrate oxidation, demonstrating that the discrepancy is not due to substrate‐uncoupled 2OG oxidation (Supporting Figure S8).

We further investigated the effects of the His to Ala substitutions on the interactions of Fe^II^ and 2OG with AspH by determining half‐maximum inhibitory concentrations (IC_50_‐values) of reported 2OG oxygenase inhibitors comprising metal ions and 2OG analogues (Supporting Information Section 2). Mn, Co, Ni, and Zn ions are reported wt AspH inhibitors^[17]^ and also inhibit both AspH variants (Table 2, entries 1–4). In agreement with results for wt AspH, most efficient AspH variant inhibition by the investigated metal ions was observed for Zn^II^, with Mn^II^ being the least potent. The reduced potency of the metal ions for AspH variant over wt AspH inhibition in part reflects the higher Fe^II^ concentration in the AspH variant inhibition assays (4 μM for H679A AspH and 10 μM for H725A AspH vs. 2 μM for wt AspH; Supporting Information Section 2). By contrast and consistent with the kinetic analyses, the inhibition results for the 2OG analogues reveal clear differences for the variants compared to wt AspH. The 2OG‐competitive broad‐spectrum 2OG oxygenase inhibitor *N*‐oxalylglycine (NOG) potently inhibits wt AspH,[^7^, ^17^] but does not apparently inhibit either AspH variant (Table 2, entry 5). This discrepancy possibly in part reflects the higher 2OG concentration used with the AspH variant, compared to the wt AspH inhibition assays (110 μM for H679A AspH and 215 μM for H725A AspH vs. 3 μM for wt AspH; Supporting Information Section 2). Another broad‐spectrum 2OG oxygenase inhibitor, pyridine‐2,4‐dicarboxylic acid (2,4‐PDCA), inhibits the AspH variants, albeit less efficiently compared to wt AspH (Table 2, entry 6). The potency of 2,4‐PDCA appears to decrease with increasing 2OG assay concentration; however, for wt AspH, it has been reported that 2,4‐PDCA maintains its potency at high 2OG concentration (IC_50_≈0.1 μM at 200 μM 2OG vs. IC_50_≈0.03 μM at 3 μM 2OG),^[18]^ suggesting that varying the AspH Fe^II^ binding ligands has a pronounced effect on the stability of the AspH:Fe^II^:2OG/2OG analogue complex.

**Table 2:** Inhibition of AspH variants by 2OG oxygenase inhibitors.

Entry	Inhibitor	AspH variant	IC_50_ [μM]^[a]^
1	MnCl_2_	wt^[17]^	5.1±1.1
		H679A	23.1±8.6
		H725A	>40
2	CoCl_2_	wt^[17]^	0.4±0.2
		H679A	1.5±0.2
		H725A	3.0±0.5
3	NiSO_4_	wt^[17]^	0.17±0.08
		H679A	2.7±0.4
		H725A	1.8±0.4
4	Zn(OAc)_2_	wt^[17]^	0.05±0.01
		H679A	0.16±0.01
		H725A	0.16±0.01
5	*N*‐oxalyl‐glycine (NOG)	wt^[17]^	1.1±0.3
		H679A	inactive
		H725A	inactive
6	Pyridine‐2,4‐dicarboxylic acid (2,4‐PDCA)	wt^[17]^	0.03±0.01
		H679A	0.13±0.01
		H725A	11.2±1.4

[a] Mean of two independent runs (*n*=2; mean ± SD). H679A/H725A AspH inhibition assays were performed as described in the Supporting Information Section 2 using 0.1 μM AspH variant and 4.0 μM hFX‐EGFD1_86‐124_‐4Ser (Supporting Figure S2a); the assays were of good quality as high S/N ratios and Z′‐factors^[19]^ (>0.5 for each plate) manifest (Supporting Figure S9).

To investigate the structural basis of the kinetic differences between wt AspH and its variants, the latter were crystallised in the presence of Mn^II^ and 2OG or NOG, with or without the synthetic hFX‐EGFD1_86‐124_‐4Ser substrate peptide. The structures were solved by molecular replacement using reported structures (PDB ID: 5JTC^[17]^ or 5JQY^[7]^) as search models. The AspH variants crystallised in one of two forms in the *P*2_1_2_1_2_1_ space group (1.6–2.7 Å resolution; Supporting Figures S10–S20 and Supporting Table S1), in accord with reported wt AspH structures.[^7^, ^8^, ^17^]

**Figure 2:**
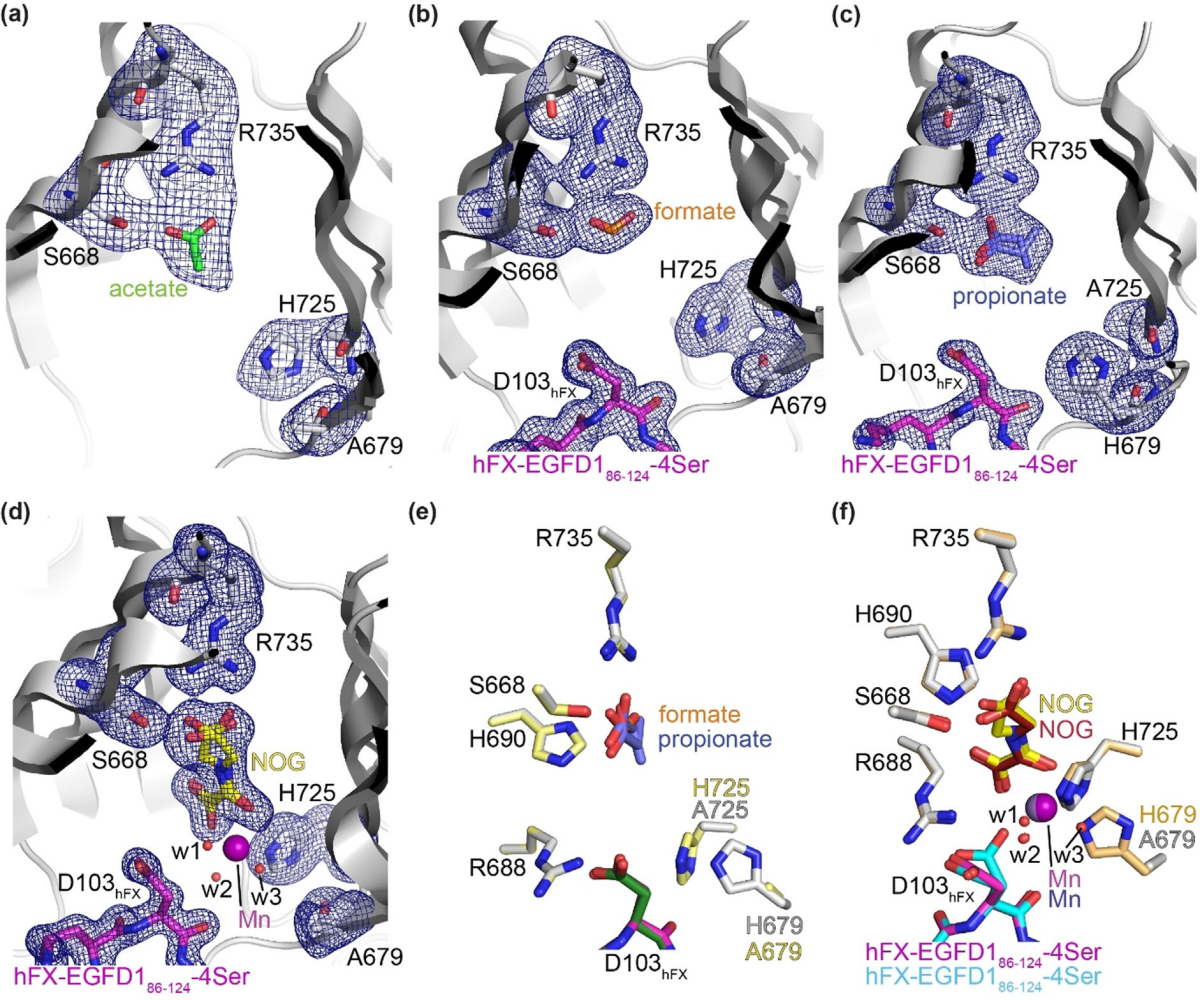
Crystal structure views of the H679A and H725A AspH variants. Colors: grey: His_6_‐AspH_315‐758_ variants; yellow: carbon‐backbone of *N*‐oxalylglycine (NOG); green: carbon‐backbone of acetate; orange: carbon‐backbone of formate; slate blue: carbon‐backbone of propionate; violet: Mn; magenta: carbon‐backbone of the hFX‐EGFD1_86‐124_‐4Ser peptide (Supporting Figure S2a); red: oxygen; blue: nitrogen. w: water. a–d) Representative OMIT electron density maps (mF_o_‐DF_c_) contoured to 3.0σ around key residues and the a) acetate in the H679A AspH:acetate structure, b) formate in the H679A AspH:hFX‐EGFD1_86‐124_‐4Ser structure, c) propionate in the H725A AspH:hFX‐EGFD1_86‐124_‐4Ser structure (note: propionate adopts two conformations and the orientation of its carboxylate differs from the orientations of acetate and formate in a,b), and d) NOG in the H679A AspH:Mn:NOG:hFX‐EGFD1_86‐124_‐4Ser structure; e) superimposition of views of the H679A AspH:hFX‐EGFD1_86‐124_‐4Ser and H725A AspH:hFX‐EGFD1_86‐124_‐4Ser structures (colours: pale yellow: H725A AspH; dark green: carbon‐backbone of the hFX‐EGFD1_86‐124_‐4Ser peptide); f) superimposition of views of the H679A AspH:Mn:NOG:hFX‐EGFD1_86‐124_‐4Ser structure and the reported wt AspH:Mn:NOG:hFX‐EGFD1_86‐124_‐4Ser structure (colours: bronze: wt AspH; lavender blue: Mn; maroon: carbon‐backbone of NOG; cyan: carbon‐backbone of the hFX‐EGFD1_86‐124_‐4Ser peptide; PDB ID: 5JQY).^[7]^

The crystallisation conditions were found to determine the nature of the AspH variant structures. When H679A AspH was crystallised with Mn^II^ and NOG, but without the hFX‐EGFD1_86‐124_‐4Ser substrate, a metal ion complex was not obtained. Comparison with a wt AspH structure reveals that the substitution of H679 by alanine neither substantially changes the overall AspH fold nor the conformation of key active site residues engaged in Fe^II^/2OG binding in wt AspH (Supporting Figure S11). Instead of the anticipated NOG, an acetate from the buffer was present at the active site, with its carboxylate positioned to interact with R735 (2.6 and 3.0 Å) and S668 (2.4 Å)—these residues normally interact with the 2OG C5 carboxylate (Figure 1 b). Several active site water molecules, but no metal ions, were observed, suggesting impaired metal ion binding capacity in the absence of the H679 imidazole (Figure 2 a).

To obtain a metal‐bound H679A AspH structure, we added the hFX‐EGFD1_86‐124_‐4Ser substrate to the Mn^II^/NOG containing crystallisation mixture aiming to stabilise metal ion binding, potentially by additional metal ion coordination with the substrate aspartyl side chain carboxylate, as observed in some cases for wt AspH:Mn:ligand:hFX‐EGFD1_86‐124_‐4Ser structures,[^7^, ^8^, ^17^] or by sterically hindering the release of the metal ion and/or 2OG/NOG. Clear electron density for EGFD1_86‐124_‐4Ser was observed in the resultant H679A AspH structure, with the AspH and substrate conformations being similar to those reported (Supporting Figures S14–S20); however, neither a Mn ion nor NOG were bound in the active site (Figure 2 b). Instead, as observed before with acetate, a formate ion from the buffer was positioned to interact with R735 (2.7 and 2.9 Å) and S668 (2.5 Å) (Figure 2 b). Similar results were obtained when H725A AspH was crystallised in the presence of Mn^II^, 2OG, and hFX‐EGFD1_86‐124_‐Ser, with a propionate ion being positioned to interact with R735 and S668, though the propionate was observed in two conformations (Figure 2 c).

These crystallographic observations correlate with the increased 2OG $K_{m}$ ‐values of the AspH variants compared to wt AspH (Table 1) and lack of inhibition by NOG (Table 2, entry 5); thus, under conditions where wt AspH crystallises with metal and 2OG/NOG (and, in some cases, hFX‐EGFD1_86‐124_‐Ser),[^7^, ^8^, ^17^] the latter are outcompeted for binding by abundant carboxylic acids in the crystallisation buffer. The crystallographic studies also unambiguously reveal the presence of only one potential Fe^II^‐binding histidine residue for the H679A and H725A AspH variants (Figure 2 a–c).

We worked to obtain crystallisation conditions in the absence of carboxylic acids in the crystallisation buffer and obtained a H679A AspH structure in complex with Mn^II^, NOG, and hFX‐EGFD1_86‐124_‐4Ser (Supporting Figure S18). The structure revealed clear density for the side chains of H725 and A679 (Figure 2 d). Superimposition of views of analogous H679A and wt AspH:Mn:NOG:hFX‐EGFD1_86‐124_‐4Ser structures (PDB ID: 5JQY)^[7]^ reveals no substantial alterations in the conformations of H725 or of residues directly engaged in NOG binding (i.e. S668, R688, H690, and R735), or of the Mn ion position (Figure 2 f).

Electron density was observed for a Mn ion, which is ligated by the NOG oxalyl group (2.1 and 2.3 Å) and three water molecules (w1: 2.2 Å, w2: 2.1 Å, and w3: 2.2 Å; Figure 2 d). As for wt AspH, the NOG C5 carboxylate group is positioned to interact with the side chains of S668 (2.5/2.9 Å) and R735 (2.2 and 2.7/3.1 Å). In addition to ligating the metal, the NOG oxalyl carboxylate is positioned to interact with the side chains of R688 (2.8 and 3.1 Å) and H690 (2.8 Å), as observed in wt AspH:Mn:NOG/2OG structures;[^7^, ^8^] however, two conformations for the NOG glycine unit are observed, one of which corresponds to the single conformation of NOG in the reported wt AspH:Mn:NOG:hFX‐EGFD1_86‐124_‐4Ser structure^[7]^ (Figure 2 f). In reported wt AspH:Mn:2OG structures, 2OG is present as a single conformer which occupies the same conformation as NOG in wt AspH structures.^[8]^ Note that wt AspH:Mn:2OG and wt AspH:Mn:2OG:substrate structures reveal that substituting NOG for 2OG has no substantial effects on the overall fold of wt AspH.^[8]^ Thus, the H679A substitution appears to disfavour NOG binding as evidenced by the two conformations observed with H679A AspH compared to the one with wt AspH, consistent with the lack of potent inhibition of the variants compared to wt AspH (Table 2, entry 5).

**Figure 3:**
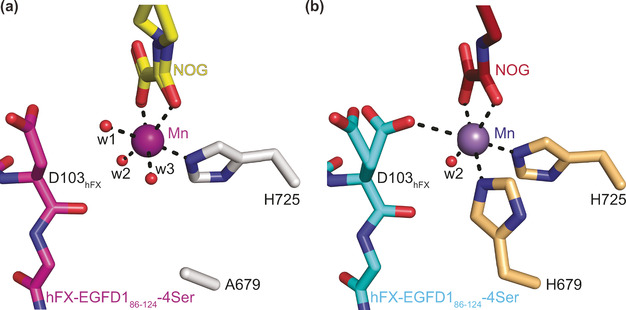
A single protein‐bound ligand (i.e. H725) supports the metal ion in the H679A AspH variant. Colors: red: oxygen; blue: nitrogen. w: water. a) Active site view of the H679A AspH:Mn:NOG:hFX‐EGFD1_86‐124_‐4Ser structure (colors: grey: H679A AspH; violet: Mn; yellow: carbon‐backbone of NOG; magenta: carbon‐backbone of the hFX‐EGFD1_86‐124_‐4Ser peptide); b) active site view of the reported wt AspH:Mn:NOG:hFX‐EGFD1_86‐124_‐4Ser structure (colours: bronze: wt AspH; lavender blue: Mn; maroon: carbon‐backbone of NOG; cyan: carbon‐backbone of the hFX‐EGFD1_86‐124_‐4Ser peptide; PDB ID: 5JQY).^[7]^

In the H679A AspH:Mn:NOG:hFX‐EGFD1_86‐124_‐4Ser complex, the Mn ion is clearly ligated by only one protein‐bound ligand, that is, H725 with the *N*τ‐imidazole‐Mn distance being 2.2 Å, the same as in wt AspH, and the A679 methyl carbon‐Mn distance being 5.8 Å (compared to 2.3 Å for the wt AspH H679 *N*τ‐imidazole‐Mn distance; Figure 2 d and 3).^[7]^ Water w2 occupies the same coordination site as the single coordinating water in the analogous wt AspH structure (Figure 3). Strikingly, water w3 occupies the coordination site of the *N*τ‐imidazole nitrogen of H679 in the wt AspH structure (Mn‐w3 distance: 2.2 Å; Figure 3), apparently compensating for the H679 to alanine mutation. Water w1 coordinates to Mn at a position *trans* to the *N*τ‐imidazole nitrogen of H725, that is, at the position where O_2_ is predicted to bind,^[8]^ in a manner which does not change the relative alignment of the substrate backbone with respect to the reported wt AspH:Mn:NOG:hFX‐EGFD1_86‐124_‐4Ser structure. A single conformation for the substrate D103_hFX_ side chain is observed in the H679A AspH:Mn:NOG:hFX‐EGFD1_86‐124_‐4Ser structure, apparently positioning its C3 methylene for stereoselective oxidation during catalysis. However, two conformations are observed for the D103_hFX_ side chain carboxylate in the wt AspH:Mn:NOG:hFX‐EGFD1_86‐124_‐4Ser structure as well as in other AspH:Mn:ligand:substrate structures, including those complexed with 2OG (Figure 3).[^7^, ^8^] In one conformation, one of the D103_hFX_ carboxylate oxygen atoms is positioned to coordinate the Mn ion (2.9 Å), that is, it occupies a position close to the position where water w1 complexes the Mn ion in the H679A AspH:Mn:NOG:hFX‐EGFD1_86‐124_‐4Ser structure. In the wt AspH:Mn:NOG:hFX‐EGFD1_86‐124_‐4Ser structure, the other D103_hFX_ side chain carboxylate conformer adopts a similar conformation as exclusively observed for the D103_hFX_ side chain carboxylate in the H679A AspH:Mn:NOG:hFX‐EGFD1_86‐124_‐4Ser structure.

## Conclusion

The combined solution studies presented here, clearly demonstrate that the H679A and H725A AspH variants retain substantial catalytic activity compared to wt AspH (Table 1 and Supporting Figure S2). The crystallographic analyses provide clear evidence for the presence of a single protein (histidine) ligand in each variant. Previous mutation studies with bovine AspH reported no activity for the H675A bovine AspH variant (corresponding to human H679A AspH by sequence alignment^[7]^), with reduced activity for the H675D/E variants, which can coordinate metals via their side chains.^[20]^

Surprisingly, the kinetic analyses revealed comparatively small effects of the H679A and H725A mutations on the $K_{m}^{\mathrm{app}}$ ‐value for Fe^II^, that is, the values for wt and H679A AspH are similar and approximately three times lower than the value for H725A AspH (Table 1). The involvement of other (second sphere) active site residues in Fe^II^ binding is likely, but there is no evidence for direct Fe^II^ chelation by other residues, though this and/or the involvement of the substrate in Fe^II^ binding cannot be entirely ruled out during catalysis. By contrast, the effects of the histidine substitutions on the $K_{m}^{\mathrm{app}}$ ‐value for 2OG are much more substantial, that is, the values for H679A AspH (≈109 μM) and H725A AspH (≈211 μM) are more than two orders of magnitude higher than the value for wt AspH (≈1.1 μM; Table 1, entry 3). In wt AspH, the H679 imidazole coordinates Fe^II^ *trans* to the 2OG C1 carboxylate (Figure 1 b and Supporting Figure S21).^[8]^ The replacement of this interaction by a water (or hydroxide) in H679A AspH may alter backdonation of electron density from the Fe ion to the ligand during 2OG binding or in intermediates during catalysis, resulting in altered kinetic parameters, including an increased H679A AspH $K_{m}^{\mathrm{app}}$ ‐value for 2OG. The H725 imidazole that coordinates Fe^II^ opposite to the proposed O_2_‐coordination site (Supporting Figure S21) may similarly regulate the stability of O_2_ (and 2OG) derived intermediates, including the Fe^III^‐2OG‐superoxide species in catalysis.

The observation of substantial catalytic activity for a 2OG oxygenase with a single protein‐based Fe^II^ ligand has implications for functional assignment work on the superfamily. The results show care should be taken in the use of Fe^II^ ligand variants in negative controls in cellular and in vivo work. Studies with isolated enzyme/substrate(s) should be carried out (note that the $K_{m}^{\mathrm{app}}$ ‐values for the AspH variants investigated are in the range of cellular 2OG concentrations^[15]^). Work with FIH has shown that substitution of the Asp‐residue of the HXD…H motif for Gly, but not Ala or Glu, enables retention of some activity (note that AspH also has an HXG…H motif), further supporting the need for experimental determination of a lack of activity.^[21]^ Secondly, bioinformatics searches for 2OG oxygenases commonly look for DSBH fold proteins with the consensus HXD/E…H motif (or an HXA/G…H motif in the case of 2OG dependent halogenases^[4]^). The knowledge that they can operate with two histidines as in the case of AspH or only one histidine ligand, substantially expands the set of potential 2OG oxygenases. Indeed, a number of potential 2OG oxygenases have atypical Fe^II^ binding residues, including, in humans, PHD finger protein 2 (PHF2)^[22]^ and hairless;^[23]^ of particular interest is the naturally occurring human AspH variant of unknown function, aspartate β‐hydroxylase domain‐containing protein 1 (AspHD1), which sequence analysis predict has only the distal His of the typical HXD/E…H motif as Fe^II^ ligand, the proximal His (i.e. corresponding to H679 in wt AspH) is substituted for an Arg residue (Supporting Figure S22). These enzymes are the subject of ongoing studies.

Many attempts to make non‐protein biomimetic oxidation catalysts of non‐heme Fe^II^ oxygenases have employed 3 or more Lewis basic metal ion ligands.^[24]^ The results presented here suggest that catalysts with 2 ligands, or even 1 ligand, should be viable—we appreciate holding the metal ion on the catalyst will be a challenge—this might be achieved by use of suitable cosubstrates (the use of cosubstrates other than 2OG has been reported to retain the catalytic activity of human 2OG oxygenases[^8^, ^25^]) or by use of metal trapping by a hydrophobic barrier as shown for other types of metal catalysts.

## Conflict of interest

The authors declare no conflict of interest.

## Supporting information

As a service to our authors and readers, this journal provides supporting information supplied by the authors. Such materials are peer reviewed and may be re‐organized for online delivery, but are not copy‐edited or typeset. Technical support issues arising from supporting information (other than missing files) should be addressed to the authors.

Supplementary
